# Active-fluidics versus gravity-fluidics in uncomplicated cataract surgery: using optical coherence tomography angiography to estimate early changes in macular and optic disc microcirculation

**DOI:** 10.1007/s00417-025-06939-x

**Published:** 2025-11-05

**Authors:** Alessandra Scampoli, Emanuele Crincoli, Lorenzo Governatori, Carlo Monaco, Cosma Danilo Mancini, Federico Giannuzzi, Matteo Mario Carlà, Giulia Grieco, Stanislao Rizzo, Tomaso Caporossi

**Affiliations:** 1 Vitreoretinal Surgery Unit, Isola Tiberina - Gemelli Isola Hospital, Rome, Italy; 2 Ophthalmology Unit, Fondazione Policlinico Universitario A. Gemelli IRCCS, Rome, Italy; 3 Catholic University of the Sacred Heart, Rome, Italy

**Keywords:** Cataract surgery, Active-fluidics system, Gravity-fluidics system, Intraocular pressure, Optical coherence tomography angiography

## Abstract

**Purpose:**

To compare, using optical coherence tomography angiography (OCTA), the earliest changes and damages to macular and optic disc microcirculation after active-fluidics system (AFS) and gravity-fluidics system (GFS) procedures in uncomplicated cataract surgery.

**Methods:**

We included 42 eyes affected by uncomplicated cataracts and divided them into two groups: 21 eyes were randomly assigned to an AFS group and 21 eyes were randomly assigned to a GFS group. Expert examiners performed OCTA 30 ± 10 min before surgery (T0), 30 ± 8 min after surgery (T1), 24 ± 2 h after surgery (T2) and 7 days after surgery (T3).

**Results:**

No significant differences at T1 were detected between the groups. At T2, eyes in the GFS group exhibited a whole macula deep capillary plexus vessel density of 37.9 ± 5.8%, which was significantly lower than that of the eyes in the AFS group (42.2 ± 5.7%; *p* = 0.048). At T3, eyes in the GFS group exhibited a significantly higher retinal nerve fibre layer thickness in the nasal (*p* = 0.020) and inferior (*p* = 0.045) quadrant, and a significantly lower peripapillary vessel density in the inferior quadrant of the papilla (*p* = 0.036) compared with eyes in the AFS group.

**Conclusion:**

The AFS procedure appears to protect macular and optic disc microcirculation during phacoemulsification, and it may represent a more prudent approach, particularly in cases that require specific care to preserve residual peripapillary and macular vasculature (e.g., diabetic or glaucomatous eyes).

## Key messages

*****What is known***:**

- The active-fluidics system reduces intraoperative intraocular pressure fluctuations, that can affect retinal and optic nerve vasculature

*****What is new***:**

- In both AFS and GFS groups, macular thickness showed a transient increase in the immediate postoperative period, accompanied by a decrease in DCP VD in both groups. It remained persistently higher in GFS-group, in parallel with a rebound increase in DCP VD with values at 7 days that were higher that baseline values.
- Seven days after surgery eyes from GFS group showed a significantly higher RNFLT in the nasal and inferior quadrant, and a significantly lower RCP VD in the inferior quadrant compared to eyes in AFS group.
- We postulated a mechanism of ischemia and reperfusion accompanied by intraretinal oedema involving both the papillary region and the macular region in GFS group.

## Introduction

In recent decades, cataract surgery has undergone important technological innovations—ranging from intracapsular and extracapsular extraction to Kelman’s phacoemulsification—that have enabled safer and faster surgeries with a shorter recovery time [1].

Most of these benefits are due to improved access to intraocular structures: in fact, phacoemulsification involves small incisions, unlike previously used techniques. Smaller incisions are associated with a lower risk of endophthalmitis and a lower risk of hypotony due to better control of intraocular pressure (IOP). A fundamental role in the regulation of IOP is played by the fluidics of the instrument used to perform the surgery. In the gravity-fluidics system, the speed and flow of irrigation is controlled by gravity, which is regulated by changing the height of the fluid by the bottle connected to the system. For example, the fluid bottle can be raised to obtain higher IOP in the case that stabilization of the anterior chamber is needed. However, using elevated pressures to avoid anterior chamber fluctuations may result in damage to ocular structures, including optic nerve vascularization, that is particularly susceptible to sudden changes in IOP.

To avoid this problem, the active-fluidics system was introduced in 2013. This fluidic system is based on constant control of IOP via two metal plates that compress a balanced salt solution, monitor perfusion flow, and ensure the stability of the anterior chamber [2]. This procedure results in reduced intraoperative fluctuations that can affect retinal and optic nerve vasculature.

We compared the impact of surgery performed using these two different types of fluidics system on macular and optic disc vasculature using optical coherence tomography angiography (OCTA).

## Materials and methods

This prospective, randomized cohort study was conducted at Gemelli- Isola Tiberina Hospital in Rome, Italy from December 2023 to February 2024. Eyes operated on uncomplicated cataract surgery were preoperatively randomly assigned to an Active-Fluidics System (AFS) group or to a Gravity-Fluidics System (GFS) group. The study followed the guidelines of the Declaration of Helsinki and all recruited patients provided written informed consent to participate in this study. Patients diagnosed with age-related, uncomplicated cataracts eligible for surgical treatment and with an age above 18-years-old were included. The exclusion criteria included diabetes mellitus, systemic hypertension and related retinopathies, high myopia, glaucoma and the presence of any coexistent ocular pathology that could affect the patient’s vision or the study’s results. Furthermore, eyes with a history of trauma, amblyopia or previous surgery (e.g., filtration surgery, vitrectomy) and eyes with an insufficient quality of preoperative acquisitions due to dioptric media opacity were excluded.

Each patient underwent a preoperative ophthalmic evaluation, including best-corrected visual acuity (BCVA) testing (decimal metrics), IOP measurements and a slit-lamp microscope examination. Opacity of the lens was classified using the LOCS III grading system [3].

All examinations were performed under the same conditions at the same location by expert examiners 30 ± 10 min before surgery (T0), 30 ± 8 min after surgery (T1), 24 ± 2 h after surgery (T2) and 7 days after surgery (T3).

OCTA imaging was performed using the RTVue XR Avanti device (Optovue Inc, Fremont, California, USA). Integrated split-spectrum amplitude-decorrelation angiography (SSADA) was used to generate the angiographic data. We followed the built-in HD AngioVue protocol 6 × 6 mm OCTA scan centred on the fovea and the 4.5 × 4.5 mm scan centred on the optic nerve head. Concerning optic nerve head status, we evaluated the following OCT and OCTA parameters: the average overall retinal nerve fiber layer (RNFL) thickness, the RNFL thickness of each quadrant (superior, inferior, nasal and temporal quadrants) and the radial peripapillary capillary plexus (RCP) vessel density (VD). Concerning macular status, we evaluated superficial capillary plexus (SCP) and deep capillary plexus (DCP) VD in the whole macula, the fovea, the parafovea and the perifovea. All considered quantitative parameters were calculated using the built-in algorithm. Images with poor signal strength (signal strength index < 6) or with motion artifacts preventing reliable image analysis were not included in the study.

### Surgical technique

All cataract surgeries were carried out by three expert surgeons (T.C., A.S. and L.G.) under topical anaesthesia. Eyes randomised to the GFS group underwent cataract surgery with Stellaris PC (Bausch + Lomb Surgical, Bridgewater, New Jersey, USA) with a bottle height at 90 cm, while eyes randomised to the AFS group were operated using Centurion Vision System (Alcon Surgical, Fort Worth, Texas, USA) with a target IOP of 40 mmHg. In all the surgeries, a Cohesive Viscoelastic (Amvisc, Bausch + Lomb Surgical) was used when appropriate during the surgical procedure. A hydrophobic acrylic intraocular lens (MX60, Bausch + Lomb Surgical) was implanted in the capsular bag in all eyes. After the surgery, the patients received dexamethasone and tobramycin eye drops to apply four times per day for two weeks and diclofenac eye drops to apply three times per day for three weeks.

### Statistical analysis

The statistical analysis was performed using SPSS software v.26 (IBM, SPSS Statistics). The normality of the quantitative variables was assessed using the Shapiro-Wilk test. We compared the quantitative variables between the groups using a two-tailed T-test for independent samples for each timepoint. Within-group analysis of the longitudinal changes was performed using one-way ANOVA for repeated measures. Qualitative variables were compared using Fisher’s exact test. A p value less than 0.05 was considered to indicate statistical significance. Bonferroni correction was applied in case of multiple comparisons.

## Results

The study included 42 eyes of 42 patients. Half of the study cohort was randomly selected to be operated using Active Fluidics System (AFS group, 21 eyes), while the rest of the population was operated using Gravity Fluidics System (GFS group, 21 eyes).

**Fig. 1:**
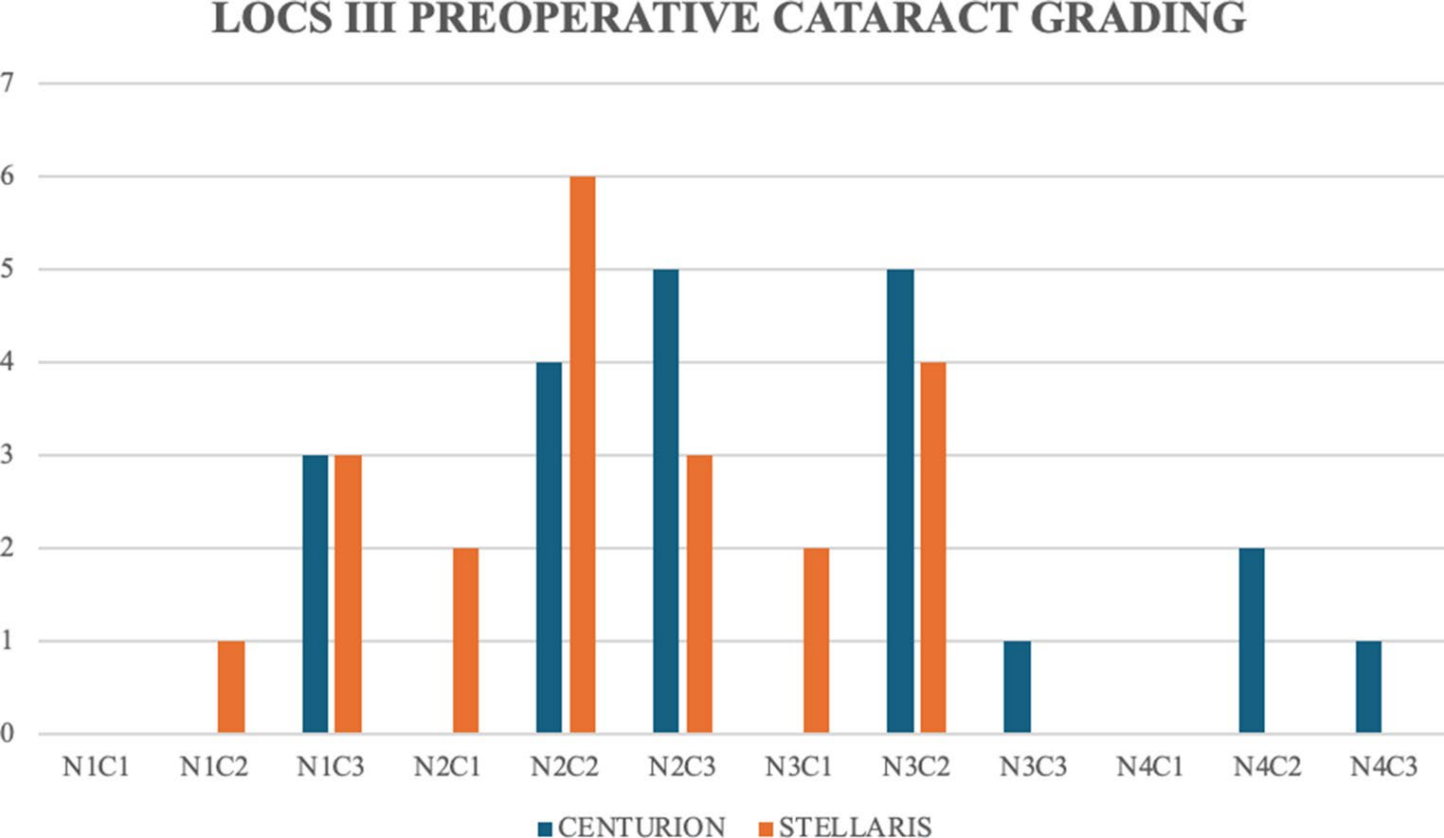
Histogram showing preoperative grading of the cataract for each group according to LOCS III grading system

No significant differences in terms of age (*p* = 0.269) or gender (*p* = 0.998) were noted between the groups (Table 1). Preoperative BCVA was 0.43 ± 0.53 decimals in the AFS group and 0.39 ± 0.52 decimals in the GFS group (*p* = 0.343). No significant difference in terms of preoperative spherical equivalent (*p* = 0.629), axial length (*p* = 0.435) or anterior chamber depth (*p* = 0.749) were noted between the groups. The LOCS III grading of the operated cataract for each group is illustrated in Fig. 1 (*p* = 0.456). No significant difference in surgical time was detected between the two study groups. No significant differences in IOP were detected between the two study groups in any of the evaluated timepoints (see Table 1).

**Table 1:** Demographic and biometric differences in the two study groups

		AFS-group	GFS-group	*p*
Age (years)		73.7 ± 7.1	76.3 ± 7.7	0.269
Male Gender		8/21 (38.1%)	7/21 (33.3%)	0.998
PreoperativeBCVA (decimals)		0.43 ± 0.53	0.39 ± 0.52	0.343
Preoperative SE (D)		-0.64 ± 1.5	-0.35 ± 1.4	0.629
Preoperative IOP		15.5 ± 1.6	14.8 ± 1.7	0.726
Postoperative IOP	T1	12.7 ± 1.6	14.2 ± 1.8	0.688
	T2	18.1 ± 2.3	19.0 ± 2.1	0.898
	T3	14.7 ± 1.9	15.5 ± 1.8	0.835
Axial lenght (mm)		23.82 ± 1.5	24.20 ± 1.5	0.435
Anterior chamber depth (mm)		3.05 ± 0.5	3.09 ± 0.5	0.749
Surgical time (min)		19.62 ± 4.3	16.67 ± 2.8	0.013*

BCVA = best corrected visual acuity; IOP= intraocular pressure; SE = spherical equivalent. * = statistically significant values

### Macular and optic nerve oedema (structural OCT)

In AFS group, a significant increase in retinal thickness was noted in the parafoveal and perifoveal regions at T1, with a return to baseline values at T3 (see Tables 2, 3 and 4). By contrast, in GFS group, the first peak of retinal thickness increase (similar to AFS group) was followed by a second peak at T3 (Table 4).

**Table 2:** Differences in baseline examination between the 2 study groups

		AFS-group	GFS-group	p
Whole macula	Retinal thickness (mm)	274.7 ± 13.2	274.2 ± 19.9	0.929
	VD SCP (%)	44.8 ± 3.9	43.3 ± 4.5	0.300
	VD DCP (%)	42.7 ± 5.1	40.8 ± 5.5	0.292
Fovea	Retinal thickness (mm)	266.2 ± 18.5	263.9 ± 41.9	0.837
	VD SCP (%)	21.2 ± 8.7	17.1 ± 7.1	0.120
	VD DCP (%)	37.2 ± 9.3	31.4 ± 7.82	0.051
Parafoveal	Retinal thickness (mm)	313.5± 13.6	315.0 ± 28.2	0.848
	VD SCP (%)	44.9 ± 5.5	43.6 ± 4.6	0.456
	VD DCP (%)	48.4 ± 4.4	47.6 ± 6.9	0.664
Perifoveal	Retinal thickness (mm)	271.2 ± 13.7	272.0 ± 21.6	0.902
	VD SCP (%)	45.3 ± 4.7	44.1 ± 5.0	0.455
	VD DCP (%)	42.6 ± 6.1	40.6 ± 5.8	0.317
Peripapillary area	RNFL S (mm)	115.5 ± 22.6	118.5 ± 15.49	0.636
	RNFL N (mm)	89.9 ± 12.3	96.7 ± 23.4	0.259
	RNFL T (mm)	74.4 ± 17.1	69.8 ± 13.4	0.348
	RNFL I (mm)	127.3 ± 27.6	128.3 ± 19.9	0.901
	RCP VD S (%)	46.9 ± 7.4	50.3 ± 7.9	0.173
	RCP VD N (%)	49.1 ± 6.9	51.3 ± 6.1	0.299
	RCP VD T (%)	46.9 ± 6.3	48.5 ± 10.2	0.582
	RCP VD I (%)	48.6 ± 6.8	50.8 ± 8.1	0.378
*DCP* deep capillary plexus, *I *inferior quadrant, *N* nasal quandrant, *RCP* radial peripapillary capillary plexus, *RNFL* retinal nervefiber layer, *S *superior quadrant, *SCP* superficial capillary plexus, *T *temporal quadrant, *VD* vessel density. * = statistically significant values

**Table 3:** Postoperative differences between the 2 study groups in 3 different timepoints: immediately after surgery (T1), 1 day after surgery (T2) and 7 days after surgery (T3)

T1				
		AFS-group	GFS-group	p
Whole macula	Retinal thickness (mm)	276.3 ± 12.6	274.5 ± 20.4	0.774
	VD SCP (%)	44.7 ± 6.7	46.4 ± 6.6	0.483
	VD DCP (%)	36.8 ± 5.8	38.3 ± 5.1	0.460
Fovea	Retinal thickness (mm)	268.5 ± 17.4	272.6 ± 44.4	0.745
	VD SCP (%)	13.7 ± 7.8	14.8 ± 8.9	0.708
	VD DCP (%)	28.9 ± 10.5	29.4 ± 13.2	0.908
Parafoveal	Retinal thickness (mm)	317.1 ± 13.1	312.2 ± 32.3	0.599
	VD SCP (%)	43.7 ± 6.4	44.7 ± 6.7	0.661
	VD DCP (%)	44.7 ± 7.8	43.9 ± 6.4	0.760
Perifoveal	Retinal thickness (mm)	272.2 ±12.9	273.3 ± 20.5	0.866
	VD SCP (%)	46.5 ± 7.6	48.3 ± 7.3	0.511
	VD DCP (%)	36.6 ± 7.3	38.8 ± 5.7	0.353
Peripapillary area	RNFL S (mm)	116.9 ± 20.6	119.3 ± 16.5	0.711
	RNFL N (mm)	88.6 ± 16.8	96.4 ± 21.8	0.271
	RNFL T (mm)	66.4 ± 9.8	76.2 ± 37.5	0.332
	RNFL I (mm)	128.9 ± 25.7	128.2 ± 20.4	0.934
	RCP VD S (%)	50.3 ± 5.1	51.4 ± 6.7	0.622
	RCP VD N (%)	49.1 ± 8.6	51.9 ± 7.8	0.338
	RCP VD T (%)	50.0 ± 6.2	50.8 ± 7.9	0.756
	RCP VD I (%)	48.2 ± 5.9	51.6 ± 10.0	0.257
T2				
		AFS-group	GFS-group	p
Whole macula	Retinal thickness (mm)	274.6 ± 15.2	273.6 ± 20.5	0.887
	VD SCP (%)	43.3 ± 4.9	44.9 ± 5.2	0.399
	VD DCP (%)	42.2 ± 5.7	37.9 ± 5.8	0.048*
Fovea	Retinal thickness (mm)	261.3 ± 17.8	264.4 ± 45.8	0.818
	VD SCP (%)	19.8 ± 8.5	15.8 ± 5.9	0.139
	VD DCP (%)	34.2 ± 10.1	29.3 ± 9.7	0.188
Parafoveal	Retinal thickness (mm)	314.8 ± 11.8	312.2 ± 30.5	0.770
	VD SCP (%)	43.2 ± 6.2	44.7 ± 5.5	0.506
	VD DCP (%)	48.8 ± 4.9	45.8 ± 6.5	0.165
Perifoveal	Retinal thickness (mm)	272.3 ± 12.5	271.9 ± 21.5	0.958
	VD SCP (%)	44.5 ± 3.9	46.3 ± 5.8	0.357
	VD DCP (%)	42.2 ± 6.5	37.5 ± 6.3	0.060
Peripapillary area	RNFL S (mm)	117.4 ± 24.9	118.8 ± 16.5	0.846
	RNFL N (mm)	89.4 ± 14.1	95.7 ± 21.9	0.327
	RNFL T (mm)	67.4 ± 13.9	66.5 ± 13.3	0.857
	RNFL I (mm)	122.3 ± 25.6	128.5 ± 18.8	0.431
	RCP VD S (%)	45.9 ± 6.7	50.2 ± 5.9	0.056
	RCP VD N (%)	48.2 ± 8.3	49.6 ± 5.9	0.178
	RCP VD T (%)	46.6 ± 5.6	49.7 ± 8.3	0.071
	RCP VD I (%)	48.6 ± 6.9	49.1 ± 5.2	0.109
T3				
		AFS-group	GFS-group	P
Whole macula	Retinal thickness (mm)	275.0 ± 12.9	276.2 ± 20.7	0.852
	VD SCP (%)	45.7 ± 4.4	44.1 ± 4.1	0.258
	VD DCP (%)	45.0 ± 5.9	42.9 ± 4.8	0.255
Fovea	Retinal thickness (mm)	263.9 ± 19.1	262.5 ± 31.4	0.872
	VD SCP (%)	20.7 ± 8.6	20.6 ± 5.7	0.980
	VD DCP (%)	36.1 ± 8.6	36.2 ± 7.3	0.946
Parafoveal	Retinal thickness (mm)	315.7 ± 13.3	312.6 ± 28.8	0.698
	VD SCP (%)	47.5 ± 5.9	45.2 ± 5.0	0.229
	VD DCP (%)	50.4 ± 5.4	49.2 ± 4.5	0.485
Perifoveal	Retinal thickness (mm)	270.9 ± 13.6	270.9 ± 16.5	0.989
	VD SCP (%)	46.1 ± 3.8	44.8 ± 4.4	0.349
	VD DCP (%)	45.7 ± 6.5	43.5 ± 5.6	0.309
Peripapillary area	RNFL S (mm)	116.8 ± 23.4	120.4 ± 15.5	0.597
	RNFL N (mm)	90.6 ± 12.8	97.8 ± 20.3	0.020*
	RNFL T (mm)	68.9 ± 13.1	69.5 ± 12.2	0.894
	RNFL I (mm)	124.6 ± 25.2	129.5 ± 17.8	0.045*
	RCP VD S (%)	47.9 ± 7.5	49.2 ± 4.0	0.532
	RCP VD N (%)	49.3 ± 7.5	48.7 ± 8.0	0.825
	RCP VD T (%)	47.5 ± 6.8	48.6 ± 4.7	0.812
	RCP VD I (%)	48.8 ± 6.8	46.9 ± 8.8	0.036*
*DCP* deep capillary plexus, *I *inferior quadrant, *N* nasal quandrant, *RCP* radial peripapillary capillary plexus, *RNFL *retinal nerve fiber layer, *S* superior quadrant, *SCP* superficial capillary plexus, *T* temporal quadrant, *VD* vessel density. * = statistically significant values				

**Table 4:** Longitudinal changes within each group

AFS-group						
		T0	T1	T2	T3	p
Whole macula	Retinal thickness	274.7 ± 13.2	276.3 ± 12.6	274.6 ± 15.2	275.0 ± 12.9	0.221
	VD SCP	44.8 ± 3.9	44.7 ± 6.7	43.3 ± 4.9	45.7 ± 4.4	0.458
	VD DCP	42.7 ± 5.1	36.8 ± 5.8	42.2 ± 5.7	45.0 ± 5.9	0.003*
Fovea	Retinal thickness	266.2 ± 18.5	268.5 ± 17.4	261.3 ± 17.8	263.9 ± 19.1	0.084
	VD SCP	21.2 ± 8.7	13.7 ± 7.8	19.8 ± 8.5	20.7 ± 8.6	0.034*
	VD DCP	37.2 ± 9.3	28.9 ± 10.5	34.2 ± 10.1	36.1 ± 8.6	0.059
Parafoveal	Retinal thickness	313.5± 13.6	317.1 ± 13.1	314.8 ± 11.8	315.7 ± 13.3	0.006*
	VD SCP	44.9 ± 5.5	43.7 ± 6.4	43.2 ± 6.2	47.5 ± 5.9	0.177
	VD DCP	48.4 ± 4.4	44.7 ± 7.8	48.8 ± 4.9	50.4 ± 5.4	0.031*
Perifoveal	Retinal thickness	271.2 ± 13.7	272.2 ±12.9	272.3 ± 12.5	270.9 ± 13.6	0.042*
	VD SCP	45.3 ± 4.7	46.5 ± 7.6	44.5 ± 3.9	46.1 ± 3.8	0.549
	VD DCP	42.6 ± 6.1	36.6 ± 7.3	42.2 ± 6.5	45.7 ± 6.5	0.007*
Peripapillary area	RNFL S	115.5 ± 22.6	116.9 ± 20.6	117.4 ± 24.9	116.8 ± 23.4	0.233
	RNFL N	89.9 ± 12.3	88.6 ± 16.8	89.4 ± 14.1	90.6 ± 12.8	0.504
	RNFL T	74.4 ± 17.1	66.4 ± 9.8	67.4 ± 13.9	68.9 ± 13.1	0.011*
	RNFL I	127.3 ± 27.6	128.9 ± 25.7	122.3 ± 25.6	124.6 ± 25.2	0.221
	RCP VD S	46.9 ± 7.4	50.3 ± 5.1	45.9 ± 6.7	47.9 ± 7.5	0.064
	RCP VD N	49.1 ± 6.9	49.1 ± 8.6	48.2 ± 8.3	49.3 ± 7.5	0.626
	RPCP VD T	46.9 ± 6.3	50.0 ± 6.2	46.6 ± 5.6	47.5 ± 6.8	0.119
	RCP VD I	48.6 ± 6.8	48.2 ± 5.9	48.6 ± 6.9	48.8 ± 6.8	0.470
GFS-group						
		T0	T1	T2	T3	p
Whole macula	Retinal thickness	274.2 ± 19.9	274.5 ± 20.4	273.6 ± 20.5	276.2 ± 20.7	<0.001*
	VD SCP	43.3 ± 4.5	46.4 ± 6.6	44.9 ± 5.2	44.1 ± 4.1	0.188
	VD DCP	40.8 ± 5.5	38.3 ± 5.1	37.9 ± 5.8	42.9 ± 4.8	0.007*
Fovea	Retinal thickness	263.9 ± 41.9	272.6 ± 44.4	264.4 ± 45.8	262.5 ± 31.4	0.190
	VD SCP	17.1 ± 7.1	14.8 ± 8.9	15.8 ± 5.9	20.6 ± 5.7	0.030*
	VD DCP	31.4 ± 7.82	29.4 ± 13.2	29.3 ± 9.7	36.2 ± 7.3	0.068
Parafoveal	Retinal thickness	315.0 ± 28.2	312.2 ± 32.3	312.2 ± 30.5	312.6 ± 28.8	0.202
	VD SCP	43.6 ± 4.6	44.7 ± 6.7	44.7 ± 5.5	45.2 ± 5.0	0.711
	VD DCP	47.6 ± 6.9	43.9 ± 6.4	45.8 ± 6.5	49.2 ± 4.5	0.015*
Perifoveal	Retinal thickness	272.0 ± 21.6	273.3 ± 20.5	271.9 ± 21.5	270.9 ± 16.5	0.098
	VD SCP	44.1 ± 5.0	48.3 ± 7.3	46.3 ± 5.8	44.8 ± 4.4	0.083
	VD DCP	40.6 ± 5.8	38.8 ± 5.7	37.5 ± 6.3	43.5 ± 5.6	0.012*
Peripapillary area	RNFL S	118.5 ± 15.49	119.3 ± 16.5	118.8 ± 16.5	120.4 ± 15.5	0.122
	RNFL N	96.7 ± 23.4	96.4 ± 21.8	95.7 ± 21.9	97.8 ± 20.3	<0.001*
	RNFL T	69.8 ± 13.4	76.2 ± 37.5	66.5 ± 13.3	69.5 ± 12.2	0.492
	RNFL I	128.3 ± 19.9	128.2 ± 20.4	128.5 ± 18.8	129.5 ± 17.8	0.003*
	RCP VD S	50.3 ± 7.9	51.4 ± 6.7	50.2 ± 5.9	49.2 ± 4.0	0.543
	RCP VD N	51.3 ± 6.1	51.9 ± 7.8	49.6 ± 5.9	48.7 ± 8.0	0.042*
	RCP VD T	48.5 ± 10.2	50.8 ± 7.9	49.7 ± 8.3	48.6 ± 4.7	0.611
	RCP VD I	50.8 ± 8.1	51.6 ± 10.0	49.1 ± 5.2	46.9 ± 8.8	0.048*
T0 = preoperative; T1 = immediately postoperative; T2 = 1 day after surgery; T3 = 7 days after surgery. *DCP* deep capillary plexus, *I* inferior quadrant, *N* nasal quandrant, *RCP* radial peripapillary capillary plexus, *RNFL* retinal nerve fiber layer, *S* superior quadrant, *SCP* superficial capillary plexus, *T* temporal quadrant, *VD* vessel density. * = statistically significant values						

Concerning the papillary area, a significant decrease in RNFL thickness in the temporal quadrant was noted between the T0 and T3 examinations in AFS group (*p* = 0.011; see Table 4).

**Fig. 2:**
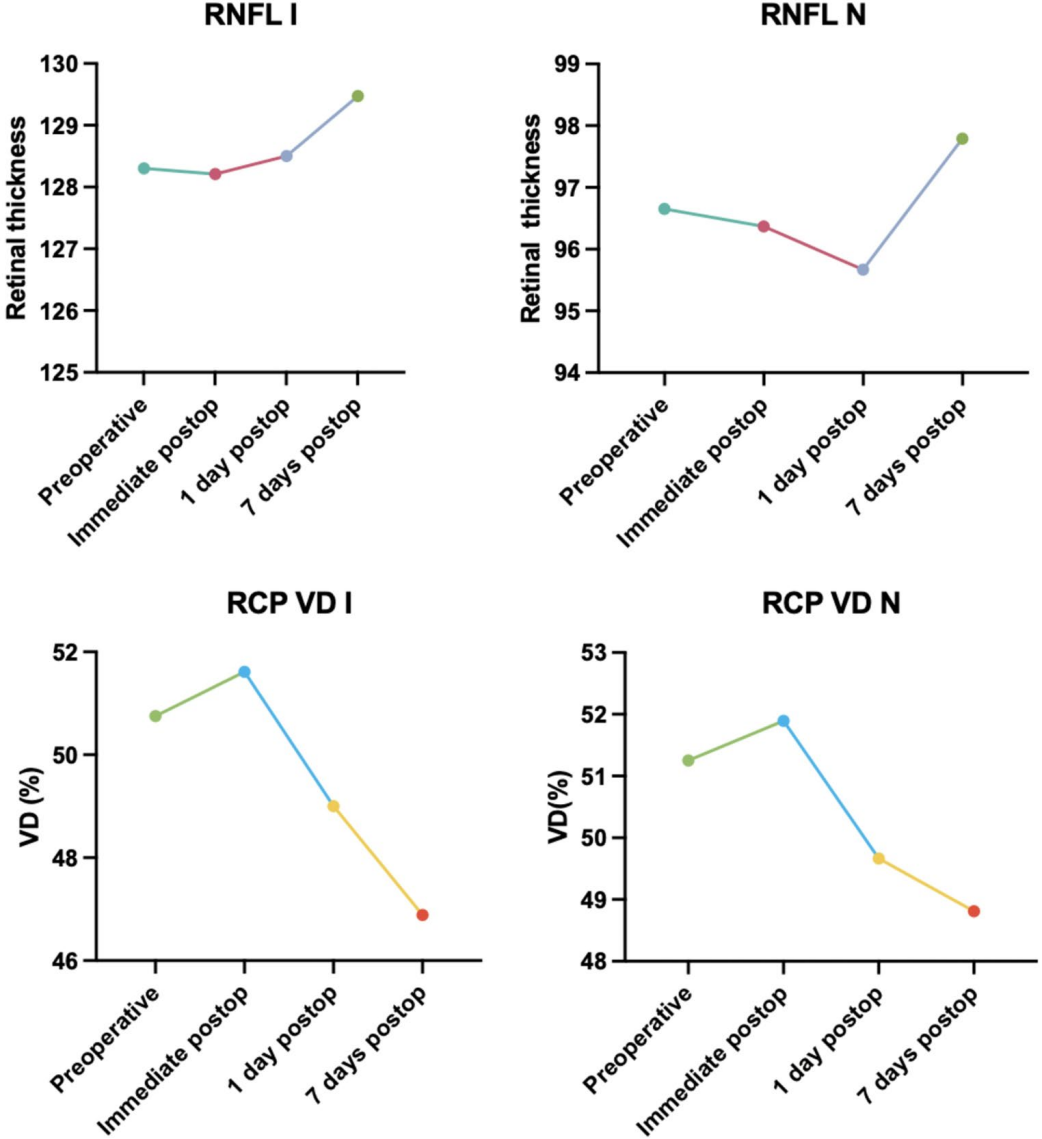
Changes in peripapillary RNFL and RCP VD in STELLARIS group during the follow up. I = inferior quadrant; N = nasal quadrant; RCP = radial peripapillary capillary plexus; RNFL = retinal nerve fiber layer; VD = vessel density

By contrast, patients in the GFS group exhibited a significant increase in RNFL thickness in the nasal and inferior quadrant at T3 (*p* < 0.001 and *p* = 0.003, respectively). Significant changes in the peripapillary area for GFS group are illustrated in Fig. 2. As a consequence, at T3, eyes in the GFS group exhibited a significantly higher RNFL thickness in the nasal (*p* = 0.020) and inferior (*p* = 0.045) compared to AFS group (Table 3).

### Capillary changes

At one day postoperatively (T2), eyes in the GFS group exhibited a significantly lower vessel density (VD) in the whole-macula deep capillary plexus (DCP) compared to those in the AFS group (see Table 3). Following a transient reduction observed immediately after surgery (T1), the AFS group showed a significant increase in DCP VD in the whole-macula, parafoveal, and perifoveal regions by one week postoperatively (T3). In contrast, the GFS group displayed a persistent decrease at T2, followed by a delayed increase at T3, with values exceeding even the preoperative levels.

In both groups, a decline in foveal superficial capillary plexus (SCP) VD was detected at T1, which normalized by T3.With respect to the peripapillary region, the GFS group showed a significant reduction in RCP VD in the same quadrants—nasal and inferior—where a concurrent increase in retinal nerve fiber layer (RNFL) thickness was observed (*p* = 0.042 and *p* = 0.048, respectively).

## Discussion

Several studies have investigated retinal microcirculation changes after cataract surgery. However, to the best of our knowledge, this investigation is the first to analyse the earliest changes in macula and optic disc microcirculation during the immediate postoperative period 1–7 days after surgery.

The innovative AFS technology integrates a pressure sensor into the phaco-handpiece to provide additional infusion fluidics to the phaco-tip during surgery to stabilise the anterior chamber and enable more physiological IOP levels during surgery. Nicoli et al. used an electronic pressure transducer (Foxboro, Honeywell) to demonstrate that the intraoperative IOP with AFS was stable at target IOP despite aspiration flow rates [4].

In terms of macular findings, macular thickness in both the AFS and GFS groups exhibited a transient increase during the immediate postoperative period. This increase was accompanied by a decrease in VD in DCP in both groups compared to their respective baseline values. However, while macular thickness returned to baseline values in the AFS group, it remained persistently higher in the GFS group. Similarly, an increase in DCP VD was noted in the GFS group, with values collected on the seventh days higher than those at baseline.

Our findings are consistent with several previous studies. Zhao et al. reported a significant increase in VD in the parafoveal region 7 days after surgery performed with GFS [5]. This increase remained unchanged at least for 3 months after surgery. Other authors have noted an increase in VD that began on the first postoperative day and persisted up to 1 month after GFS surgery [6]. Similarly to our findings, Zhou et al. noted a transient decrease in macular thickness one day postoperatively but an increase in thickness 7 days postoperatively [6]. Zhao et al. directly compared the results of GFS and AFS surgery 1 month postoperatively and detected a significant increase in SCP VD and retinal thickness after GFS surgery [7]. These authors concluded that AFS protected retinal vasculature after cataract surgery. Previous studies have reported that cataract surgery might induce inflammatory reactions due to a release of cytokines that trigger a progressive increase in retinal thickness that peaks one month after surgery [5–8]. Some authors have suggested that posterior segment inflammation following anterior surgical injury leads to the rupture of the blood-retinal barrier in both inner and outer retinal layer and later in the choroid [9–11]. That situation may be responsible for the increase in macular thickness and volume compared with preoperative levels. Nevertheless, the exact mechanism and pathogenesis behind retinal changes after phacoemulsification surgery need to be investigated.

We speculated that this postoperative reduction in DCP may be related to early ischemic damage due to high intraoperative IOP that led to intracellular oedema of the macula with an increase in macular thickness. This mechanism is similar to what was previously described by Romano et al. [12]. These authors reported a transient postoperative inflammatory increase in RNFL thickness in cases of combined surgery with poor postoperative visual improvement. In our opinion, the reduction in DCP and the ischemic oedema that resolved faster with AFS are the result of this technique’s improved IOP control and stable values during surgery. In this context, the later increase in VD DCP in the GFS group can be regarded as an inflammatory vasodilation occurring during reperfusion, similar to the vasodilatation occurred in postischemic reperfusion syndrome described in cardiovascular diseases.

In terms of our peripapillary findings, eyes in the GFS group exhibited a significantly higher RNFL thickness in the nasal and inferior quadrant and a significantly lower RCP VD in the inferior quadrant 7 days after surgery compared with eyes in the AFS group. We conclude that AFS appears to protect the RCP and RNFLT from the ischemic damage. We postulated a mechanism of ischemia and reperfusion accompanied by intraretinal oedema involving the papillary region similar to what was noted above for the macular region.

Regarding optic disc microcirculation, previous studies have reported that RNFLT increased after cataract surgery [7, 13]. Zhou et al. noted that the increase in both RNFLT and mean GCL thickness initiated earlier than the changes in the entire retinal thickness [6]. She et al. reported that RNFL thickness was positively related to VD in healthy patients and that both were the highest in superior and inferior sectors [14]. Zhu et al. found no significant correlation between VD changes and postoperative RNFL thickness after cataract surgery [13]. Karabulut et al. reported an increase in VD in the total, inside-disc, and peripapillary ONH regions one month after cataract surgery but noted no differences between values at baseline and those obtained at the one-week follow-up [15]. However, the absence of significant RNFL swelling in the immediate postoperative period appears to contradict the inflammatory hypothesis underlying the observed changes. This discrepancy might be attributed to the limited sample size. We prudently selected patients with mild-to-moderate cataracts to avoid affecting the quantitative measurements of OCTA imaging; we also selected an OCTA signal strength above 6 to guarantee images of acceptable quality. Since recent studies have reported no significant correlation between changes in retinal vasculature and cumulative dissipated energy, total aspiration time and estimated fluid usage, we did not consider these parameters in our statistical analysis [7].

In conclusion, while increases in IOP are typically temporary, the long-term impact of elevated IOP could surpass perfusion pressure and consequently lead to a decrease in ocular blood flow and potentially harm the ONH [13]. Despite the small sample size of our investigation, our prospective study suggests that AFS may represent a more prudent approach and should therefore be preferred, when possible, particularly in cases that require specific care to preservation of residual peripapillary and macular vasculature (e.g., diabetic or glaucomatous eyes).
